# Effect of Two Brands of Glaze Material on the Flexural Strength and Probability of Failure of High Translucent Monolithic Zirconia

**DOI:** 10.3390/ma14227022

**Published:** 2021-11-19

**Authors:** Raj Gaurav Singh, Kai Chun Li, Karl Michael Lyons, John Neil Waddell

**Affiliations:** Department of Oral Rehabilitation, Faculty of Dentistry, University of Otago, Dunedin 9016, New Zealand; KC.li@otago.ac.nz (K.C.L.); karl.lyons@otago.ac.nz (K.M.L.); neil.waddell@otago.ac.nz (J.N.W.)

**Keywords:** high translucent, monolithic, zirconia, flexural strength, fractography

## Abstract

(1) Background: The effect of glazing on the mechanical properties of monolithic high translucent zirconia is not well reported. Therefore, the purpose of this study was to evaluate the effect of glazing on the flexural strength of high translucent zirconia; (2) Methods: Ninety specimens were prepared from second-generation 3Y-TZP high translucent blocks and divided into three groups. Glaze materials were applied on one surface of the specimen and subjected to a four-point bending test and flexural stress and flexural displacement values were derived. Descriptive fractographic analysis of surfaces was conducted to observe the point of failure and fracture pattern.; (3) Results: Control-nonglazed (647.17, 1σ = 74.71 MPa) presented higher flexural strength values compared to glaze I (541.20, 1σ = 82.91 MPa) and glaze II (581.10, 1σ = 59.41 MPa). Characteristic strength (σ_Ɵ_) from Weibull analysis also observed higher (660.67 MPa) values for the control specimens. Confocal microscopy revealed that glazed surfaces were much rougher than control surfaces. Descriptive fractographic analysis revealed that there was no correlation between the point of failure initiation and flexural strength; (4) Conclusions: The test results demonstrated that glazing significantly decreased the flexural strength and flexural displacement of the zirconia specimens.

## 1. Introduction

As a restorative material, yttria-stabilised tetragonal zirconia polycrystal (Y-TZP) is widely used to fabricate highly aesthetic and strong dental restorations using CAD-CAM technology [1,2]. In the past, several studies have reported high fracture rates from 6 to 15% on conventional/first-generation 3Y-TZP frameworks veneered with ceramics over a period of 3–5 years [3,4,5,6]. This cause was found to be a result of the high residual stress built up within the porcelain layer of the material after firing and has been resolved by the introduction of a slow cooling thermal cycle at the final stage of production [7]. Despite the issue being remedied, the issue of a weaker porcelain layer ultimately pushed 3Y-TZP to be developed as a monolithic restoration to overcome problems related to the chipping of veneered porcelain [8,9]. Monolithic 3Y-TZP is an excellent material choice due to its superior mechanical properties, but the main drawback is the low translucency, which limits the aesthetic appearance. The introduction of second-generation highly translucent monolithic 3Y-TZP (3 mol% yttrium oxide and <0.1 wt.% alumina) has gained popularity for producing highly aesthetic dental restorations [10,11,12,13]. The high translucency of 3Y-TZP is achieved by altering grain size, increasing the percentage of cubic phase and reducing alumina content [14]. Nowadays, second-generation high translucent monolithic 3Y-TZP restorations are widely used due to their superior mechanical properties, biocompatibility, resistance to corrosion and aesthetics [1,15]. In the pursuit of better aesthetic outcomes, third-generation zirconia (4Y-PSZ/5Y-PSZ) was introduced which was metastable in the tetragonal phase and contained up to 53% of the cubic phase. Doping of 4 mol%/5 mol% of yttrium oxide and reducing of alumina content (<0.1 wt.%) provides this partially stabilised zirconia with a mixed cubic/tetragonal structure [16,17]. Since the cubic structure has more volume compared to the tetragonal, light scatters less strongly at grain boundaries, providing better translucency and according to the manufacturer, Tosoh Corporation, as cited by Stawarczyk et al., no hydrothermal ageing occurs in third-generation zirconia and the material retains its strength and microstructure increasing its wear resistance [11,12,13]. The main disadvantage of this generation of PSZ is that the cubic/tetragonal stabilisation results in lower fracture toughness of third-generation zirconia. Therefore, the most translucent third-generation zirconia materials were announced for anterior restorations and limited to 3-unit bridges in the posterior regions [18,19,20].

Glazing, accompanied by staining of ceramics is a common step in dental laboratories to achieve the final shade and finish of ceramic restorations. The aim is to seal the open pores on the surface of sintered porcelain and to produce a smooth glossy layer [21]. Despite there being several reports that glazing monolithic zirconia wears off the surfaces in contact with antagonists [22,23,24], the practice of staining and glazing monolithic restorations are commonly employed to achieve an improved and acceptable aesthetics that is superior to that of the monolithic intrinsic colour of the crown [21,25,26].

Manawi et al. observed that glazed zirconia samples had significantly higher flexural strength and fracture toughness compared to ground, polished and finished zirconia [27]. However, several other studies found that glazing does not significantly improve the strength of ceramics [28,29,30]. Yener et al. and Kumchai et al. compared different zirconia materials and found that glazing reduced the flexural strength of specimens [31,32]. Asli et al. conducted a study to observe the effect of grinding and glazing on pre-sintered zirconia blocks and found that grinding followed by glazing slightly decreased the flexural strength, but the decrease was not statistically significant [33]. In a similar study, the effect of different surface alterations on flexural strength of monolithic zirconia showed that the mean flexural strength of the reglazed group was significantly higher [34]. These studies used either three-point bending, which will produce higher flexural strength values [35] or biaxial flexural strength tests, which are dominated by internal defect initiation of failure [25], in their methodology. A preferable alternative is four-point bend flexural testing, which is based on the principle that the test captures a larger portion of the stress concentration of the specimen between two inner rollers and that this area is subjected to a constant bending moment as compared to the three-point bending test, thereby avoiding premature failure [36].

These previous studies focused on first- and second-generation zirconia with sample sizes of *n* = 10. These lower sample sizes limit the ability to report valid results using Weibull probability of failure statistics [37]. In addition, no fractographic analysis was carried out to establish the source of the failure which enables the fractographic determination of the location and cause of crack initiation in the failed brittle components. The effect of glazing on flexural strength of monolithic high translucent zirconia is not well reported and requires further investigation with larger sample sizes using Weibull and fractographic analysis. Therefore, the purpose of this study was to evaluate the probability of failure of two glaze systems on the flexural strength of high translucent 3Y-TZP using a four-point bending test. The null hypothesis of the study was that there will be no significant difference (*p* < 0.05) in the flexural strength between the glazed and unglazed second-generation high translucent 3Y-TZP. 

## 2. Material and Methods

### 2.1. Specimen Preparation

High translucent 3Y-TZP blocks (VITA YZ^®^ HT, VITA Zahnfabrik, Bad Säckingen, Germany) were used in the study (Table 1). A total of 90 zirconia specimens (3.75 mm × 4.95 mm × 32 mm) were sectioned from zirconia blocks using a sectioning machine under dual nozzle water irrigation (Acutom-50, Struers, Ballerup, Denmark). The cuts were made using a diamond-impregnated blade (M1D13, Struers) at 3200 rpm at a feed speed of 0.120 mm/s. Specimens were prepared according to the guidelines provided by ISO 6872:2015 for ceramic materials. The zirconia specimens were then polished under water irrigation using 1200-grit silicon carbide abrasive paper to achieve a 15 μm to 20 μm surface roughness. The corners of the specimens were bevelled (45° edge chamfer) in a polishing machine (TegraPol-21, Struers). Specimens were inspected under the microscope (Nikon LV 150, Tokyo, Japan) for the presence of the 45° edge chamfer after the bevelling. Specimens were polished prior to sintering to avoid potential phase transformation from post-sintering polishing.

All zirconia specimens were sintered in a furnace (VITA ZYRCOMAT, VITA Zahnfabrik) according to the manufacturer’s instructions (Table 2). Shrinkage of zirconia (~19%) was taken into consideration before and after sintering to achieve the final dimensions of specimens (3 mm × 4 mm × 30 mm) as per ISO 6872:2015. Specimen dimensions were verified using a digital calliper (Mitutoyo, Aurora, IL, USA) and all specimens were ultrasonically cleaned in water for 2 min to remove any contaminants from handling and then dried with compressed air. Specimens were stored in a plastic container at room temperature.

### 2.2. Glazing of Specimens

Thirty zirconia specimens were randomly selected for each sample group, which consist of one sample group of non-glazed zirconia and two sample groups of glazed zirconia. Celtra universal overglaze paste and liquid (Celtra universal overglaze, Dentsply, York, PA, USA) and IPS Empress universal glaze paste (IPS Empress universal glaze paste, Ivoclar Vivadent, Amherst, MA, USA) were used for glazing the specimens. Glaze materials were applied on one surface of each specimen and glaze firing was done in a dental furnace (Programat^®^ P500, Ivoclar Vivadent) as per the manufacturer instructions (Table 3). Control specimens were also treated under the same dry thermal glaze firing cycle as the Celtra universal glaze to eliminate any error induced by the higher firing temperature on the mechanical properties of specimens.

Specimens were designated according to the surface treatments as Group CP/Control (non-glazed), Group DG/Glaze I (Celtra universal overglaze paste and liquid) and Group IG/Glaze II (IPS Empress universal glaze paste).

### 2.3. Flexural Strength Test

**Figure 1:**
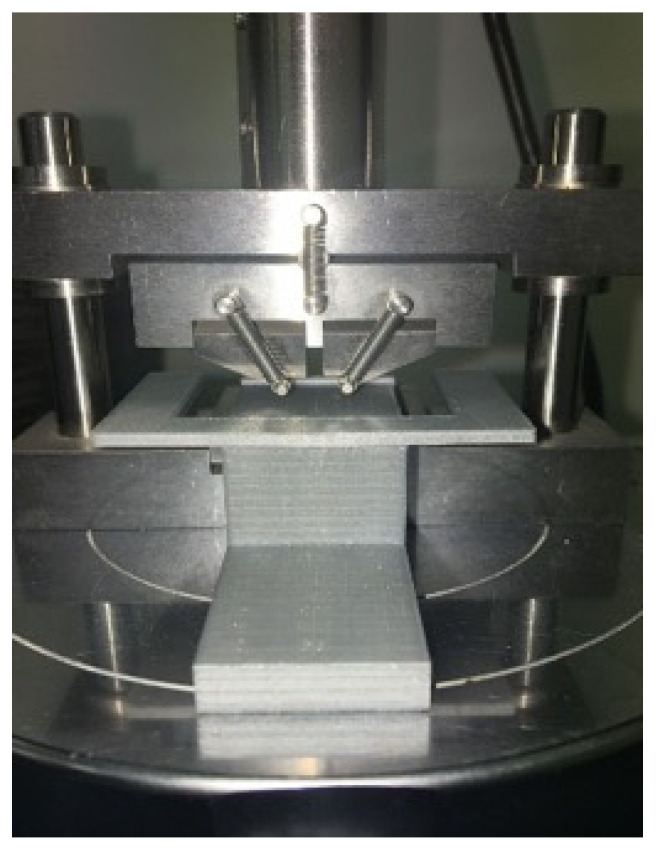
3D printed alignment jig positioning the specimen in the centre of the four-point bending jig.

A universal testing system for compressive and tensile strength (Model 3369, INSTRON^®^, Norwood, MA, USA) was used to conduct the four-point bend test using a self-aligning four-point bend jig (Flexural strength of ceramics test fixture ASTM C 1161, configuration A, fixture number WTF-CF-43, Wyoming Test Fixtures, MA, USA). A custom-made jig was 3D printed (Ultimaker S5, Utrecht, Netherlands) and used to position the specimens in the centre of the four-point bend device which were then loaded to failure using a 5kN load cell at a crosshead speed of 1.0mm/min (Figure 1). The glazed surfaces were placed on the tension side of the four-point bend test jig. Flexural strength and flexure displacement at maximum load were recorded using BlueHill^®^ Universal software (Version 4.08, INSTRON^®^). Each specimen was measured prior to testing and the measurements were entered into the testing software that calculated the flexural strength. Flexural strength was calculated using the following formula:

(1) $$\sigma =\frac{3PL}{4wb^{2}}$$

where

*σ* is flexural strength, in megapascals;
*P* is the load at failure, in newtons;
*L* is the centre-to-centre distance between outer support rollers, in millimetres;
*w* is the width of the specimen, i.e., the dimension of the side at right angles to the direction of the applied load, in millimetres;
*b* is the thickness of the specimen, i.e., the dimension of the side parallel to the direction of the applied load, in millimetres.

### 2.4. Scanning Electron Microscopy/Energy-Dispersive X-ray Spectroscopy (SEM/EDX)

Three specimens from each sample group (low fracture value, middle fracture value and high fracture value) were sputter-coated with gold/palladium for electric conductivity (Polaron E5000, Quorum Technology, Laughton, UK). SEM images were obtained at a magnification of 100×, 200× and 500× using an accelerating voltage of 10kV to examine surface characteristics (JEOL 5410LV SEM, JEOL, Hertfordshire, UK).

One specimen from each group was carbon-coated and EDX was conducted for elemental analysis of glazed and unglazed specimens (JEOL 5410LV SEM, JEOL). Three random areas were selected on each specimen to compare the results. In built Excel, tools were used for data interpretation. 

### 2.5. Confocal Microscopy—Surface Roughness Test

Three specimens from each group were randomly selected for confocal laser scanning microscopy (CLSM) observation. Specimens from each group were individually placed on the stage of a confocal microscope (Zeiss Axio imager Z.2, Oslo, Norway) with the treated surface upright and scanned. The reflection image of the surface was generated using an Ar/Ar Kr laser, with an objective magnification of 20× and numerical aperture set at 0.8 (20×/0.8 M27), selecting a scan format of 424.7 µm × 424.7 µm. The stage was moved vertically (*z*-axis) and a series of 22 optical sections were generated at 1.45 µm intervals. The scan speed, laser and optical lens magnification were the same for measuring all the specimens during the study. Fiji software (GPL v2) was used for qualitative and quantitative analysis of the following roughness parameters:

Average roughness (*Ra*) defined as the arithmetic average of the profile ordinates within the measured section (average height) [38,39].
Root mean square roughness (*Rq* or RMS) defined as the root mean square value of the profile ordinates within the measured section [38,39].
Valley roughness (*Rv*) defined as the depth of the deepest valley in the profile (based on the average height) [38,39].
Peak roughness (*Rp*) defined as the maximal height of the profile ordinates (based on the average height) [38,39].

### 2.6. Fractography

Three specimens of high, medium and low flexural strength were selected from each group for fractographic analyses. Specimens were sputter-coated with gold/palladium (Polaron E5000, Quorum Technology) and fracture patterns of zirconia specimens were observed at different magnifications and 10kV accelerating voltage using a scanning electron microscope (JEOL 5410LV SEM, JEOL UK) to establish the point of initiation of failure.

### 2.7. Weibull Analysis

The reliability of materials was evaluated using Weibull analysis and linear regression (LR) analysis was used to interpret the results. As previously described by Quinn and Quinn the usual method was followed to regress the ln ln [1/(1 − *Pf*)] values onto ln (fracture stress), or in other words, to minimise the vertical discrepancies in the graph. The slope of the line represents the Weibull modulus, *m*. The characteristic strength, σ*_Ɵ_*, is the amount of stress for which ln ln [1/(1 − *Pf*)] is zero, or *Pf* = 63.2% [37].

### 2.8. Statistical Analysis

Normal distribution of data was tested, and descriptive analysis was formed using Kolmogorov–Smirnov and Shapiro–Wilk tests. One group in the flexural strength data and all three groups in the flexural displacement data showed deviation from the normal distribution. Data were further analysed using Kruskal–Wallis and Dunn’s post hoc test. The significance level was set to *p* < 0.05 for flexural strength and flexural displacement data interpretation. Significance values in between the groups were also adjusted by the Bonferroni correction for multiple tests. The statistical analysis was performed using IBM SPSS Statistics v25 (IBM Corp., Armonk, NY, USA).

## 3. Results

**Figure 2:**
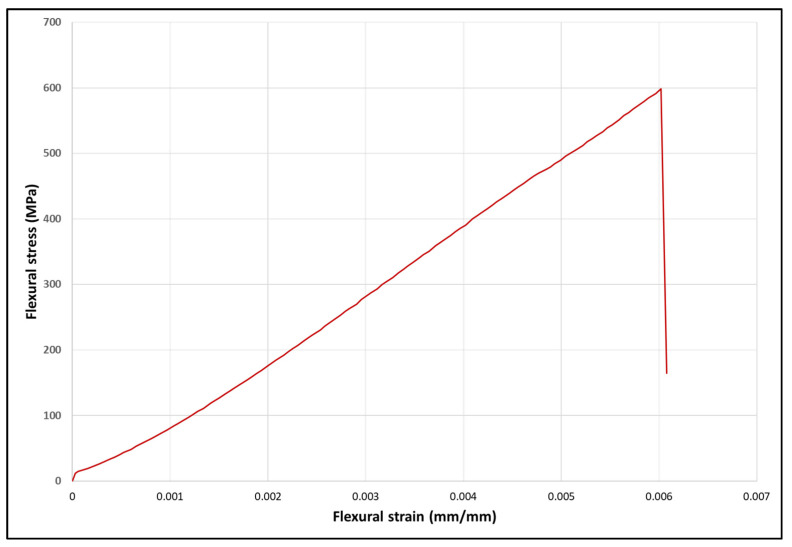
Typical Stress/Strain plot for a specimen.

**Figure 3:**
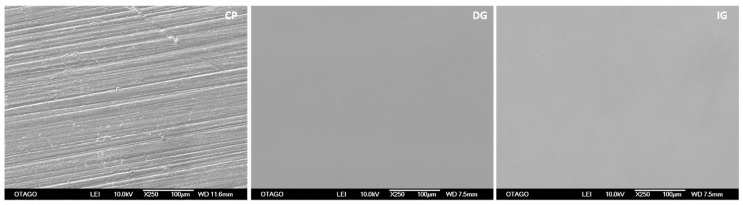
SEM images showing surface texture at 250× (group CP, DG and IG). Note the nonglazed surface appear rougher compared to glazed surfaces.

The glazed specimens showed a mean increase in thickness of 3.02 mm and 3.01 mm for DG and IG respectively compared to the control of 3 mm. Glazing reduced the flexural strength and flexural displacement of the specimens. There was a significant decrease (*p* < 0.001) in flexural strength between unglazed (control) and glazed groups (Table 4). Similarly, flexural displacement showed a significant decrease (*p* < 0.00001) between unglazed and glazed specimens. There were no statistical differences between the glazed groups. All the specimens showed a typical stress-strain plot for brittle material (Figure 2). At 250× magnification, the SEM images showed the glazed surfaces to be smoother than the control surfaces. A few defects were observed on the glazed surfaces, but generally, the surfaces appeared to be defect-free (Figure 3).

Energy-dispersive X-ray spectroscopy confirmed the elements present in the materials used to produce the specimens was the same as the information provided by the manufacturer. The amount of silica, alumina and oxides were found in higher concentrations on glazed surfaces due to the basic composition of glazes (Table 5). Zirconia traces were not found in glazed specimens confirming uniform coating of the glaze layer.

**Figure 4:**
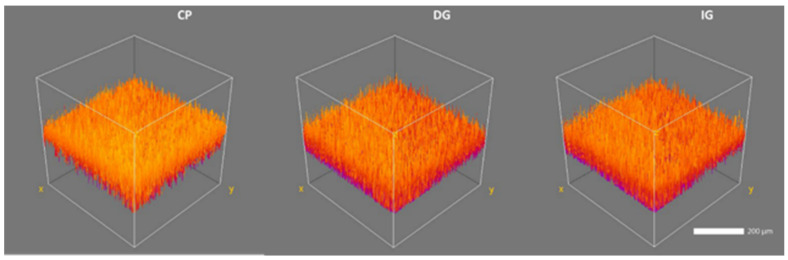
Three-dimensional representative CLSM images of specimens (group CP, DG, IG). Note the surface texture, crest and valley patterns of the specimen surfaces.

Confocal microscopy revealed the glazed surfaces were rougher when compared to the control/polished surfaces. The mean *Ra*, *Rq*, *Rv* and *Rp* values are presented in Table 6. Three-dimensional representation of control and glazed surfaces revealed the surface morphology, crest and valley patterns on each surface (Figure 4).

**Figure 5:**
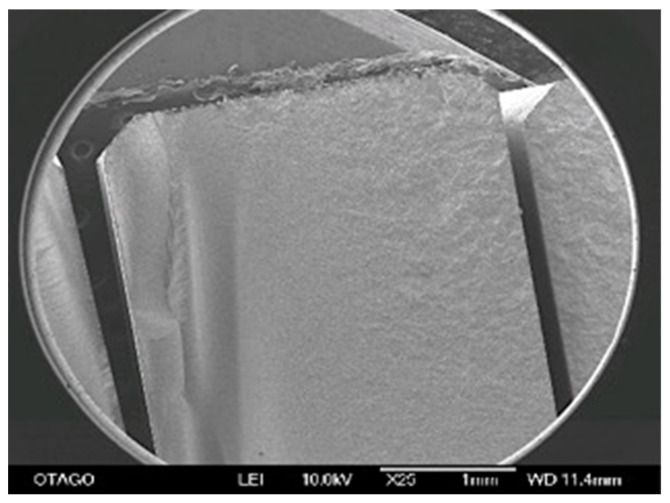
SEM images showing an example of fracture pattern at 25× magnification (group CP). Note the more course fractured surface for highest flexural strength specimens.

**Figure 6:**
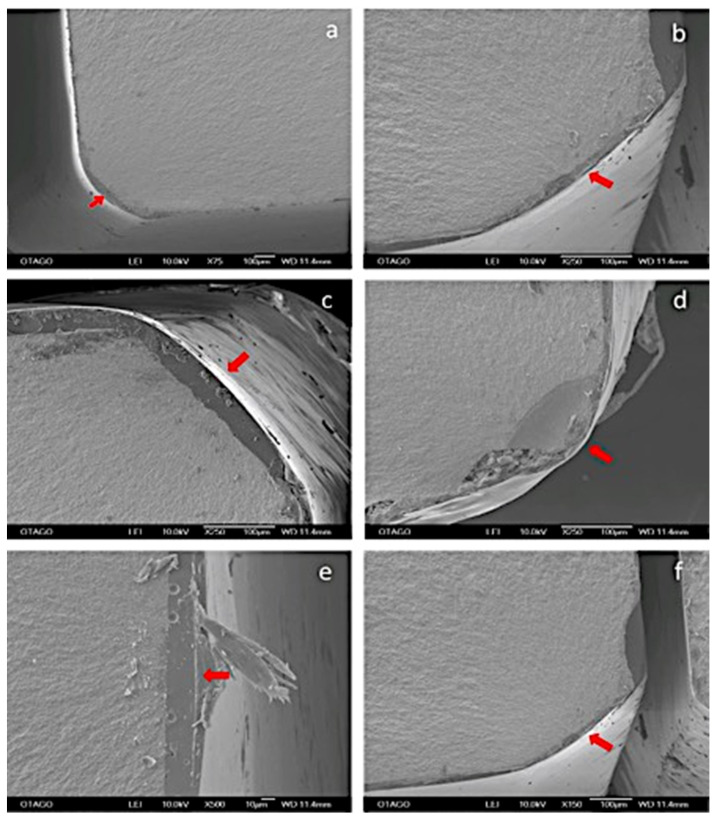
(**a**–**f**) SEM images show an example of a fracture pattern at different magnifications. The red arrow shows a point of fracture initiation and direction of crack propagation through the glaze layer.

**Figure 7:**
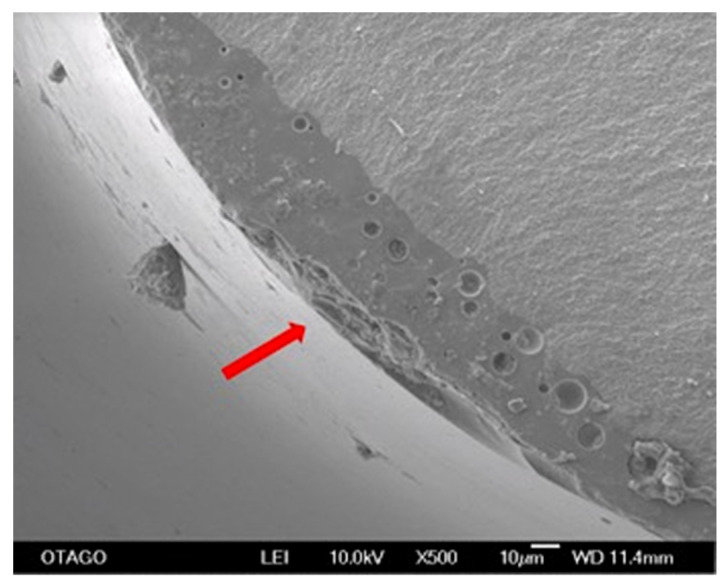
SEM image showing the presence of voids in the glaze layer and a red arrow showing the direction of crack propagation at 500x magnification (group DG).

Fractographic analysis showed a coarser fractured surface with specimens with high flexural strength (Figure 5). From the SEM images, there appeared to be no correlation between the point of failure initiation and flexural strength. Fracture surfaces showed a typical mirror pattern [40] from point of initiation (Figure 6). Glazed specimens showed the presence of voids in the glaze layer at the point of crack initiation (Figure 7).

**Figure 8:**
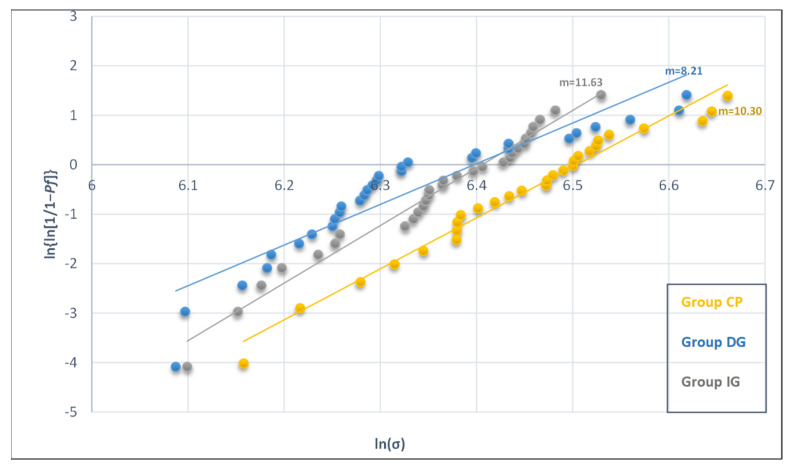
Weibull plot for flexural strength of group CP, DG and IG.

Weibull analysis confirmed the reliability of the previous experiments (Figure 8) by showing that Weibull modulus readings (group CP = 10.30, group DG = 8.21 and group IG = 11.63) were above 5 [37,41]. Control samples (unglazed) had the highest characteristic strength (660.67 MPa) compared to group DG (600.42 MPa) and group IG (605.35 MPa) (Table 7).

## 4. Discussion

In this study, the effect of two glaze systems on the flexural strength of high translucent monolithic zirconia was tested and compared with unglazed specimens. Results showed that glazing of high translucent monolithic zirconia resulted in a significant decrease (CP = 647.17, 1σ = 74.71 MPa, DG = 541.20, 1σ = 82.91 MPa and IG = 581.10, 1σ = 59.41 MPa) in flexural strength. There was no significant difference (*p* < 0.05) between the two glaze materials, therefore, the null hypothesis is that there will be no significant difference (*p* < 0.05) in the amount of flexural strength between the glazed and unglazed zirconia was rejected.

Manawi et al. tested flexural strength and fracture toughness of In-Ceram zirconia core material after glazing, grinding, finishing and polishing, and found that glazing increased the flexural strength of zirconia core material [27]. Three different disc-shaped zirconia materials were tested in another study and found that glazing significantly reduced the flexural strength of zirconia [32]. A three-point bending test was conducted on three high translucent zirconia bars and found that glazing decreased the flexural strength of specimens [31]. The effect of two glaze methods (spray and brush) was studied on translucent Y-TZP and it was suggested that glaze application methods did not damage the fatigue strength of the tested Y-TZP [42]. Previously it was found that glaze on the tensile side decreased the flexural strength, whereas glaze on the compression side was similar to unglazed specimens [43]. However, these results were based on three-point bending or biaxial flexural strength tests. The thickness of the glaze layer could have been another possible reason for these different results. In our study, we have measured the thickness of the specimens after the application of the glaze layer and this was found negligible. A thicker layer should increase the flexural strength in theory, yet the glaze had the effect of decreasing the flexural strength in the present experiment.

The use of a four-point bending test used in our study was based on the principle that the four-point bending test captures a larger portion of the specimen between two inner rollers and that this area is subjected to a constant bending moment as compared to the three-point bending test [36]. In addition, Bitter et al. demonstrated that the four-point bending test is better suited to testing the effects of surface layer additions as opposed to the biaxial-bend test, in that the biaxial-bend test showed that internal defects linking to surface defects were the dominant mode of failure whereas four-point bend tests were dominated by surface defects near or at the edge of the bend bars [25]. This suggests that the biaxial-bend test may not correctly evaluate the effect of surface defects, in that one can assume the number and size of internal defects would be the same for all the test groups despite the addition of a very thin glaze layer [35]. For brittle materials like ceramics, flexural strength depends on both their toughness and the size of the surface defect. When a larger volume of the specimen is exposed to the maximum stress, it reduces the measured flexural strength because it increases the probability of having cracks reaching critical length at a given applied load. Flexural strength values measured with four-point bending tests are significantly lower than the three-point bending test [44]. For these reasons, we believe that the results of our study can be considered as having a high level of validity and therefore can more accurately be extrapolated to the clinical situation. This also explains why our results are different to those published by the manufacturer who used a three-point bend test and other studies which also used three-point bending and biaxial flexural testing.

There are a limited number of studies on the effect of glazing on the flexural strength of high translucent zirconia and many of these studies used ten or fewer specimens per sample group which limit the statistical reliability in terms of reporting Weibull probability of failure. A good rule of thumb is that a minimum of 30 specimens be tested, as adhered to in the present study, to provide adequate Weibull strength distribution parameters, with more test specimens contributing little towards better uncertainty estimates [37].

The Weibull modulus (m) is a shape parameter, where higher m values indicate that the fracture stress values are close to each other and will enable better prediction of the expected strength of a material [37]. Thus, the Weibull modulus is generally referred to as the factor which gives the reliability of the material [37]. In our present study, the Weibull modulus for all specimen groups were all found to be above 5 (Figure 8), which indicates it meets the minimum threshold for good reliability based on the criteria proposed by ISO 6872:2015. Furthermore, our results showed that unglazed specimens had a higher characteristic strength (660.67 MPa) than glazed specimens (Table 7).

SEM was used in this study to inspect the fracture origin, fracture pattern and path of crack propagation. The strength of a brittle material is dependent upon the size of the biggest defect present [37]. In the present study, SEM observation showed that glazing appeared to lead to a smooth fracture surface when compared with unglazed surfaces. A few odd defects were observed, but the glazed surfaces were defect free and did not appear to influence the fracture initiation. Although most of the specimens fractured from the corner, there were some variations in the site of fracture initiation. Of note, we used a self-aligning four-point bend jig which ensured that any potential misalignment of a specimen was not exacerbated using a fixed bending jig thereby inducing the failure at an artificially induced high-stress area. In a previous fractographic study, pores were observed in the glaze layer, but these did not seem to initiate fracture [45]. We also observed voids in the glaze layer at the glaze/zirconia interface at the corner of several specimens which may have caused fracture initiation at those sites (Figure 7). The voids in the glaze layer could have been the result of a mismatch of coefficient of thermal expansion of monolithic zirconia and glaze layer during cooling. An investigation that was conducted on the time and temperature of sintering of porcelain on porosity formation, suggested that porosities were minimum at high temperatures and shorter periods of time [46]. They also found that the formation of porosities in the dentine is more temperature-dependent. Some previous studies used SEM and surface profilometer for qualitative and quantitative analysis of surface roughness after glazing and different polishing techniques. Interestingly, all the surfaces showed partial cracks and porosity except glazed surfaces and there was no correlation between SEM and surface profilometry [47]. In our study, the surface of the glaze layer appeared smoother than the control surfaces at 250× magnification and failure was initiated from these surfaces.

While the low magnification (250×) SEM observation suggested that glazed surfaces were much smoother than the control, CLSM revealed the opposite (Figure 4). When the *Ra* and *Rq* values were compared, the glazed surfaces were rougher than the control surfaces (Table 6). A previous study comparing glazed and polished ceramic surfaces using CLSM, also found that glazed ceramic specimens were rougher compared to polished specimens [39]. This study also suggested that the presence of large porosities on the glaze surface led to a rougher surface compared to the polished bulk material. In our study, the difference in resolution and scanning between the CLSM and the low magnification SEM was that the CLSM was able to identify/quantify the surface topography/roughness more accurately at the micro surface level and thereby quantify the potential defects that would lower the flexural strength of the glazed specimens compared to the control group.

Manawi et al. stated that the rougher the surface, the lower the flexural strength and fracture toughness [27]. In our study, we observed that a more course fractured surface was associated with specimens with higher flexural strength values (Figure 5). Both the flexural displacement and flexural stress of the glazed groups was also significantly reduced (Table 4) because of the glaze (which was applied to the tension side of the specimens). A likely explanation of this, based on the high-resolution SEM images, was that the brittle glaze layer assisted in the initiation of fracture more so than the control group and this high fracture energy then extended into the 3Y-TZP at lower stress values than the control. CLSM results showed that glaze surfaces were rougher than unglazed zirconia. This suggests that the surface defects/flaws were deeper, thus a crack can more easily propagate spontaneously on this surface. SEM analysis of fractured specimens also showed that the crack initiation from the glaze to zirconia occurred seamlessly through the interface (Figure 6 and Figure 7), supporting the theory that the glaze allowed spontaneous crack propagation to occur more easily due to deeper surface flaws. This build-up of the fracture energy to propagate in the glaze extends to the zirconia, even if it’s not enough to fracture zirconia, it may be enough to create a defect, which then weakens the zirconia and the overall flexural strength.

Previous studies [27,31,32,33,34] reporting on the effect of glaze on the flexural strength of Y-TZP did not mention how many surfaces they applied the glaze layer to, only stating that specimens were "glazed according to manufacturer’s instructions". If glaze is applied to the compression side of the test specimen, it would potentially increase the flexural strength. In contrast, if the glaze layer is applied only to the tension side of the test specimen, it has the potential of weakening the flexural strength of the system as was the result observed in our study. If the glaze is applied to both the tension and compression sides of the specimen, then this may cancel out the effect with no increase or decrease in flexural strength. Without clear identification of where the glaze is applied to the specimen, it is difficult to directly compare our results with previous studies. A recent study by Lobo et al. reported the effect of glaze application and mechanical ageing on the biaxial flexural strength of Y-TZP and found that glaze applied on both the tensile and compression sides improve the flexural strength of Y-TZP [45]. This study, however, only used a sample size of *n* = 10 on which to report their Weibull results.

In this in vitro study, specimens were shaped according to the guidelines provided by ISO 6872:2015 for ceramic materials and glazed only on one surface which underwent tension during flexural strength test to simulate clinical conditions where crowns are glazed on their outer surface. Apart from one other study [31] which used beams to measure the flexural strength, all the others [27,32,33,34] used the biaxial flexural strength piston-on-three-ball method to measure the flexural strength. This makes it problematic to compare our results with theirs. Although in vitro flexural strength tests can never replicate the actual geometry of crowns, further studies are needed to evaluate clinically shaped restorations under simulated thermal and mechanical oral conditions to compare the results with these results. A limitation of our study was that we were unable to source the Vita brand of glaze material for inclusion in the study due to supply chain problems at the commencement of our study. An additional limitation of our study was that we used the second generation of zirconia as opposed to the latest third generation zirconia. The reason for this was that 4Y-PSZ/5Y-PSZ are evolving in terms of super/extra translucent zirconia, but the main limitation is their reduced strength. As a next stage research theme, new generations of zirconia (4Y-PSZ/5Y-PSZ) should be tested for the mechanical properties, optical properties and fracture mechanics.

## 5. Conclusions

Glazing significantly decreased the mean flexural strength and flexural displacement of the zirconia specimens as well as the characteristic strength from the Weibull analysis. There was no statistically significant difference between the flexural strength of the two brands of glaze applied to the glazed zirconia specimens.

No correlation was observed between the fracture initiation site and the flexural strength of high translucent second-generation 3Y-TZP. However, a continuous fracture from the initiation site at the glaze through to the 3Y-TZP was observed in all specimens, indicating that the glaze layer weakens the fracture resistance of 3Y-TZP.

As a result of this finding, and within the limitations of the study, we would recommend that in high aesthetic areas where characterisation is needed to achieve clinically acceptable results and bite forces are lower, glazing with stains is recommended, whereas, in the posterior/non-aesthetic regions where bite forces are higher, polishing of monolithic zirconia without glazing is recommended.

## Figures and Tables

**Table 1:** Brand name, manufacturer and chemical composition of the material tested.

Brand name	Manufacturer	Composition
VITA YZ^®^ HT	VITA Zahnfabrik, Bad Säckingen, Germany	ZrO_2_ wt.%: 90.4–94.5, Y_2_O_3_ wt.%: 4–6, HfO_2_ wt.%: 1.5–2.5, Al_2_O_3_ wt.%: 0–0.3, Er_2_O_3_ wt.%: 0–0.5 Fe_2_O_3_ wt.%: 0–0.3

**Table 2:** Sintering parameters of zirconia specimens.

Duration of the program cycle including cooling down to 200 °C: approximately 7.5 h
Rising time: 1.5 h
End temperature: 1530 °C
Holding time at end temperature: 2 h
Cooling down to 400 °C with firing chamber being closed

**Table 3:** Glaze firing protocol used for two glaze materials and the control group.

Glaze Material	Drying Time (min)	Pre-Heat (min)	Low Temp (°C)	Heat Rate (°C/min)	High Temp (°C)	Vac Start Temp (°C)	Vac Stop Temp (°C)	Hold Time Vac (min)	Hold Time Air (min)	Cool Time (min)
Control 3Y-TZP	2	2	500	55	820	0	0	0	1.30	3
Celtra universal overglaze	2	2	500	55	820	0	0	0	1.30	3
IPS Empress universal glaze paste	2	2	403	100	789	0	0	0	1.30	6

**Table 4:** Flexural strength and flexural displacement median values with standard deviation.

Group	Flexure Strength (1σ) (MPa)	Flexural Displacement at Maximum Flexure Stress (1σ) (mm/mm)
CP	647.17 (74.71)	0.015 (0.009)
DG	541.20 (82.91)	0.010 (0.005)
IG	581.10 (59.41)	0.008 (0.003)

**Table 5:** Mean value (wt.%) of elements present on the surfaces on control and glazed specimens.

Elements	Mean wt.% in Group CP	Mean wt.% in Group DG	Mean wt.% in Group IG
Zr	70.30	-	-
O	23.76	45.8	45.78
Y	4.16	-	-
Al	0.06	5.94	3.35
Si	-	27.69	33.92
Hf	1.72	-	-
Na	-	6.35	5.86
Mg	-	1.36	-
K	-	4.34	5.76
Ca	-	1.07	1.02
Ti	-	0.68	0.73
Ba	-	5.78	-
Ce	-	0.99	0.48
Zn	-	-	2.68

**Table 6:** The mean surface roughness values (µm) with standard deviation.

Group	Mean *Ra* (SD)	Mean *Rq* (SD)	Mean *Rv* (SD)	Mean *Rp* (SD)
CP	1.45 (0.11)	1.88 (0.16)	−3.4 (0.37)	5.30 (1.68)
DG	1.63 (0.11)	2.07 (0.09)	−3.48 (0.49)	6.96 (1.39)
IG	2.28 (0.13)	2.94 (0.27)	−5.54 (1.26)	7.91 (0.43)

**Table 7:** Table showing characteristic strength (calculated failure stress), Weibull modulus and mean coefficient of variation of group CP, DG and IG groups. Note the control group shows the highest characteristic strength.

Group	Characteristic Strength (MPa) Defined as Approx. 63.2%	Weibull Modulus (m)	Mean Coefficient of Variation (%)
CP	660.67	10.30	29.75
DG	600.42	8.21	14.65
IG	605.35	11.63	10.25

## Data Availability

The data presented in this study are available on request from the corresponding author. The data are not publicly available due to privacy.
